# sFlt-1/PlGF Ratio as a Predictive Marker in Women with Suspected Preeclampsia: An Economic Evaluation from a Swiss Perspective

**DOI:** 10.1155/2019/4096847

**Published:** 2019-08-14

**Authors:** Markus Hodel, Patricia R. Blank, Petra Marty, Olav Lapaire

**Affiliations:** ^1^Obstetric Clinic, Lucerne Cantonal Hospital, Spitalstrasse, 6000 Luzern 16, Switzerland; ^2^Roche Diagnostics (Switzerland) AG, Industriestrasse 7, 6343 Rotkreuz, Switzerland; ^3^Department of Obstetrics, University Hospital Basel, Spitalstrasse 21, 4031 Basel, Switzerland

## Abstract

In Switzerland, 2.3% of pregnant women develop preeclampsia. Quantification of the soluble fms-like tyrosine kinase-1 (sFlt-1) and placental growth factor (PlGF) ratio has shown a diagnostic value in the second and third trimesters of pregnancy, in particular in ruling out preeclampsia within one week. We estimated the economic impact of implementing sFlt-1/PlGF ratio evaluation, in addition to the standard of care (SOC), for women with suspected preeclampsia from a Swiss healthcare system's perspective. A decision tree model was developed to estimate direct medical costs of diagnosis and management of a simulated cohort of Swiss pregnant women with suspected preeclampsia (median week of gestation: 32) until delivery. The model compared SOC vs. SOC plus sFlt-1/PlGF ratio, using clinical inputs from a large multicenter study (PROGNOSIS). Resource use data and unit costs were obtained from hospital records and public sources. The assumed cost for sFlt-1/PlGF evaluation was €141. Input parameters were validated by clinical experts in Switzerland. The model utilized a simulated cohort of 6084 pregnant women with suspected preeclampsia (representing 7% of all births in Switzerland in 2015, *n* = 86,919). In a SOC scenario, 36% of women were hospitalized, of whom 27% developed preeclampsia and remained hospitalized until birth. In a sFlt-1/PlGF test scenario, 76% of women had a sFlt-1/PlGF ratio of ≤38 (2% hospitalized), 11% had a sFlt-1/PlGF ratio of >38-<85 (55% hospitalized), and 13% had a sFlt-1/PlGF ratio of ≥85 (65% hospitalized). Total average costs/pregnant woman (including birth) were €10,925 vs. €10,579 (sFlt-1/PlGF), and total costs were €66,469,362 vs. €64,363,060 (sFlt-1/PlGF). Implementation of sFlt-1/PlGF evaluation would potentially achieve annual savings of €2,105,064 (€346/patient), mainly due to reduction in unnecessary hospitalization. sFlt-1/PlGF evaluation appears economically promising in predicting short-term absence of preeclampsia in Swiss practice. Improved diagnostic accuracy and reduction in unnecessary hospitalization could lead to significant cost savings in the Swiss healthcare system.

## 1. Introduction

Preeclampsia is a disorder of pregnancy, defined as the onset of hypertension, proteinuria, or other maternal organ dysfunctions after 20 weeks of gestation [1, 2]. It occurs in 2.3% of pregnancies in Switzerland and 2-8% of pregnancies globally [3, 4]. Accurate, timely diagnosis of preeclampsia is crucial to reducing maternal and fetal perinatal morbidity and mortality and preventing long-term postnatal maternal complications [4, 5]. However, diagnosis of preeclampsia is often complicated by a heterogeneous disease course and historic absence of sensitive and specific diagnostic tests [6].

Maternal circulation of proangiogenic and antiangiogenic biomarkers is altered in preeclampsia [7]. In particular, studies have shown that the ratio of soluble fms-like tyrosine kinase-1 (sFlt-1) to placental growth factor (PlGF) is elevated in preeclampsia and is raised even before clinical onset of the disease [7, 8]. This ratio has been used successfully in clinical trials to improve prediction of preeclampsia for women at risk of this condition [9, 10]. The sFlt-1/PlGF ratio showed better predictive ability compared with using a single parameter (e.g., PlGF alone) [11].

The PRediction of short-term Outcome in preGNant wOmen with Suspected preeclampsia Study (PROGNOSIS) is a previously reported, international, multicenter, prospective, double-blind, noninterventional study [10, 12]. PROGNOSIS was the first clinical study to evaluate the sFlt-1/PlGF ratio as a tool for short-term prediction of preeclampsia in pregnant women with suspected preeclampsia. Using the fully automated Elecsys® sFlt-1 and PlGF assays (Roche Diagnostics) for maternal blood testing, cut-offs for the sFlt-1/PlGF ratio were derived and validated to rule out (for up to 4 weeks; sFlt-1/PlGF ratio ≤ 38) or rule in (within 4 weeks; sFlt-1/PlGF ratio > 38) the occurrence of preeclampsia [10, 13]. Retesting women with suspected preeclampsia 2 or 3 weeks after their initial test was also shown to improve risk stratification for preeclampsia [13]. These data suggest that the use of the sFlt-1/PlGF ratio may enable better patient management for women with suspected preeclampsia, as clinicians can identify low- and high-risk patients and ensure that they are managed appropriately. This may help to reduce unnecessary hospitalization and extended monitoring and thereby be cost saving for the healthcare system.

Previously, Vatish et al. (2016), Schlembach et al. (2018), and Frusca et al. (2017) published economic assessments of the use of the sFlt-1/PlGF ratio for the prediction of preeclampsia in the UK [14], Germany [15], and Italy [16], respectively. Patient-level data were taken from the PROGNOSIS study, and country-specific economic models were developed. The authors concluded that introducing the sFlt-1/PlGF ratio into clinical practice for women with suspected preeclampsia would result in cost savings of £344 per patient in the UK, €671 per patient in Italy, and €361 per patient in Germany, mainly by improving the ability to rule out preeclampsia and thereby reducing unnecessary hospitalization. Utilizing clinical data from the PROGNOSIS study [10, 12] and Swiss-specific economic data, this analysis evaluated the economic impact of using the sFlt-1/PlGF ratio, in addition to standard of care (SOC), for the short-term prediction of preeclampsia in a simulated study cohort of pregnant women with suspected preeclampsia from a Swiss healthcare system's perspective [17].

## 2. Materials and Methods

### 2.1. Model Structure

We developed an Excel-based decision tree model (cost-effectiveness analysis) to estimate the direct medical costs of diagnosis and management of pregnant women with suspected preeclampsia (median week of gestation: 32), including delivery, based on the assumption suspected and manifest preeclampsia that occurs in 7% of all pregnancies [18]. The model simulated the progression of a cohort of pregnant Swiss women by assessing the risk of developing preeclampsia and the consequent decision to hospitalize or to manage the women in an outpatient setting, centering on ruling out preeclampsia. Expected costs were compared between the testing and no-testing strategies from the perspective of the Swiss healthcare system. The patient pathway was based on the PROGNOSIS study [10, 12] and adapted to patient management protocols in Switzerland. The average weekly costs of patient management were included in the model, based on clinical data derived from the PROGNOSIS study (including false positive/negative results). Unit costs were derived from the registries of two Swiss hospitals (Basel University Hospital (BUH) and Lucerne Cantonal Hospital (LCH)) and official Swiss tariffs (Tarmed v.1.09 and Analysenliste v.2.01) [19, 20]. Monetary values are presented in euros (converted from CHF at an exchange rate of 1 CHF = €0.88, September 2018).

### 2.2. PROGNOSIS Study

The PROGNOSIS study was a large multicenter, prospective, noninterventional study that evaluated serum sFlt-1/PlGF ratios in 1050 pregnant women with suspected preeclampsia between 24 weeks and 36 weeks plus 6 days of gestation [10, 12]. An sFlt-1/PlGF cut-off of 38 was derived and validated for use in the short-term prediction of preeclampsia, using the Elecsys® sFlt-1 and PlGF electrochemiluminescence immunoassays [10, 12]. During PROGNOSIS, both clinicians and participants were unaware of the sFlt-1/PlGF status due to the double-blind study design. Therefore, all treatment decisions were made in the absence of this knowledge [10, 12]. Data on the sFlt-1/PlGF ratio, diagnosis, and resource use (including planned/unplanned hospital visits and inpatient length of stay) were recorded [12]. Details of ethics approval and patient consent have been reported previously for PROGNOSIS [10, 12].

### 2.3. Patient Groups

**Figure 1:**
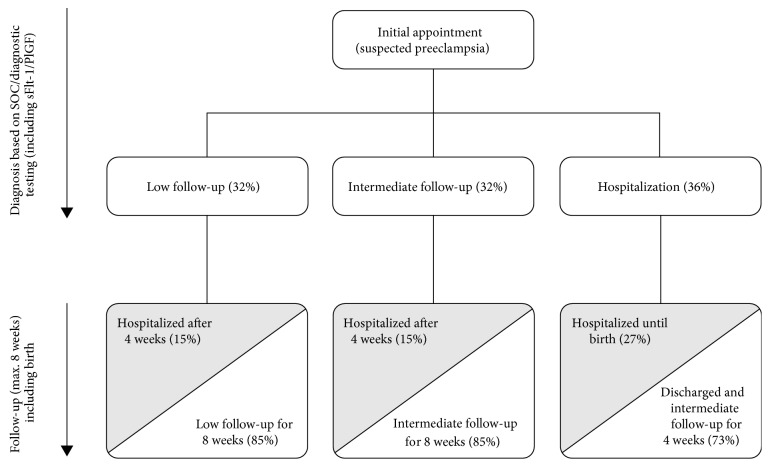
Patient management diagram for the no-testing strategy (based on standard of care) and assumed hospitalization rates according to the PROGNOSIS study. Grey shading indicates confirmed cases of preeclampsia. PlGF: placental growth factor; sFlt-1: soluble fms-like tyrosine kinase-1; SOC: standard of care.

**Figure 2:**
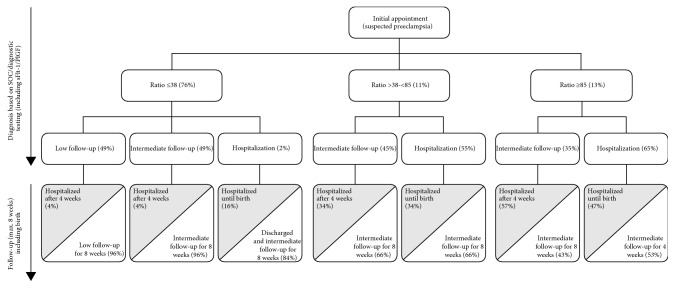
Patient management diagram for the testing strategy (sFlt-1/PlGF ratio determined) and assumed hospitalization rates according to the PROGNOSIS study. Grey shading indicates confirmed cases of preeclampsia. PlGF: placental growth factor; sFlt-1: soluble fms-like tyrosine kinase-1; SOC: standard of care.

The patient population for this study consisted of pregnant women with suspected preeclampsia, defined as the onset of proteinuria and hypertension after 20 weeks of gestation, without specified diagnosis of preeclampsia [12]. In the current model, we estimated that 7% of all pregnancies would be the suspected (unconfirmed) cases of preeclampsia, based on published guidance [18, 21]. Therefore, 6084 simulated pregnancies were included in the analysis (representing 7% of all births in Switzerland in 2015, total *n* = 86,919). In the no-testing scenario, outpatient management (low/intermediate) and hospitalization rates for women with suspected preeclampsia were estimated following an initial consultation, according to SOC (Figure 1). In the testing scenario (SOC and sFlt-1/PlGF), outpatient management (low/intermediate) and hospitalization rates were simulated according to the sFlt-1/PlGF ratio (≤38, >38-<85, or ≥85; Figure 2). There are many possible reasons why a clinician may decide that a woman with a sFlt-1/PlGF ratio of ≤38 should be hospitalized; however, it is not possible to quantify all possible scenarios from the PROGNOSIS data. Based on available quantifiable data, for the purpose of this model, it was assumed that a woman would be hospitalized if her blood pressure exceeded 160/110 mmHg. In the overall PROGNOSIS population, 2% of women had a sFlt-1/PlGF ratio of ≤38 with blood pressure > 160/110 mmHg.

### 2.4. Resource Use in Outpatient Management

At present, there are no specific guidelines available on how women with suspected preeclampsia should be treated in an outpatient setting. Therefore, clinical management was based on guidance provided by two clinical experts (O. Lapaire and M. Hodel) from Switzerland. Following an initial consultation, all women in the outpatient setting were assigned to low or intermediate follow-up services for a maximum duration of 8 weeks. These patients could be hospitalized after 4 weeks if their symptoms worsened (Table 1). Low or intermediate follow-up services included regular consultations, blood pressure measurements, prenatal ultrasound including Doppler, cardiotocography, and proteinuria testing for both groups, plus additional blood analyses. After 4 weeks, women with sFlt-1/PlGF ratios of ≥85 could change to intermediate follow-up or inpatient management (Figure 2).

### 2.5. Resource Use in Inpatient Management

Women hospitalized due to suspected preeclampsia were treated in one of two ways: (1) hospitalized (without birth) due to suspected preeclampsia, then discharged after a few days if preeclampsia did not manifest, or (2) hospitalized due to preeclampsia with birth (with potential lung maturation at <34 weeks of gestation; vaginal birth or C-section). Women without preeclampsia were also hospitalized due to birth (vaginal birth or C-section). In Switzerland, inpatient costs are based on diagnosis-related groups (DRGs); as such, all services are covered within one DRG position [22].

### 2.6. Unit Costs and Cost Analyses

Unit costs for outpatient care (including the initial consultation and low and intermediate follow-up settings) were derived from the official Swiss tariff list (available at http://www.tarmed-browser.ch/de). Tarmed is the official unified tariff list used in ambulatory care to charge for medical services. The Tarmed tariff positions are multiplied by a tariff value, which differs among cantons in Switzerland. In our model, the Tarmed tariff value of €0.78 (mean value for all Swiss cantons in 2016) was used as a reference. Costs of medical services provided in both the low and intermediate follow-up settings are presented in Table 2. It was assumed that patients in the low setting would receive an initial consultation and six additional follow-up consultations within 8 weeks. For the intermediate setting, we simulated clinical management for an average patient, including the medical and diagnostic services listed for ambulatory care at LCH. All assumptions were validated by clinical experts (O. Lapaire and M. Hodel).

Unit costs for the inpatient setting were derived from two Swiss hospitals: LCH, Lucerne, Switzerland, and BUH, Basel, Switzerland (Table 3). The LCH and BUH hospitals are similar in size, comprising 856 and 773 total patient beds, respectively, and 36 and 45 obstetric beds in the women's health units. We conducted a search within the accounting departments of LCH and BUH to determine the actual cost of managing patients with suspected and manifested preeclampsia for 2016. The same methodology and search filters were used for both hospitals. Cases from the obstetric clinics of both hospitals were included in the present study if coded according to a list of preset search terms (see Supplementary Table 1 for the full list). An additional search was conducted for patients with hypertension; however, no relevant results were found.

Patients (*n* = 301) included within this cost analysis were divided into three groups: (1) patients with suspected preeclampsia who did not give birth (*n* = 36), (2) patients with suspected preeclampsia who had vaginal births (*n* = 101), and (3) patients with suspected preeclampsia who gave birth via C-section (*n* = 164). The cost of a vaginal birth or C-section without preeclampsia was assessed based on DRG codes and official tariffs and the assumption that one-third of all pregnant women in Switzerland give birth via C-section [23]. The cost of one sFlt-1/PlGF analysis was calculated to be €141, including material, instrumental, and labor costs. Budget impact analysis was conducted to assess the effects of the testing strategy on the Swiss healthcare system over the course of 5 years. The costs were discounted by 3.5%.

### 2.7. Sensitivity Analyses

Sensitivity analyses were conducted to test the robustness of our results across different scenarios, including variation in hospitalization and test costs (±20%) and variations in the proportion of women hospitalized depending on the sFlt-1/PlGF ratio. For the latter, variations included the proportions of women hospitalized with a ratio of ≥85 or >38-<85, varied by ±20%. An sFlt-1/PlGF ratio cut-off of ≥85 was selected as it has previously been shown to be of value for confirming diagnoses of preeclampsia [11]. The relatively low hospitalization rate of women with an sFlt-1/PlGF ratio of ≤38 (2%) was varied by –20% and +100% to increase the robustness of our model. Additionally, the effect of the inclusion of a retest was analyzed for three different scenarios: one based on a retest rate of 6.5% derived from data in the Preeclampsia Open Study (PreOS) for women in the low outpatient setting (i.e., if the initial test was negative, ratio ≤ 38); one based on a 100% retest rate where every woman was retested irrespective of the sFlt-1/PlGF ratio; and one based on 4 times retesting of all intermediate follow-up patients (for diagnosis and prognosis) [24, 25]. In addition, a scenario analysis was conducted where birth costs were excluded.

## 3. Results

### 3.1. Patient Groups

In Switzerland, there were 86,919 pregnant women or births in 2015 [21]. Of these, it was assumed (based on published guidance) that 7% were patients with suspected preeclampsia [18, 21]. Therefore, a simulated cohort of 6084 pregnant women was included in the present analyses. In the no-test scenario, 36% of women were admitted to a hospital, 27% of whom went on to develop preeclampsia and remained hospitalized until birth (Figure 1). In the test scenario (i.e., where information on the sFlt-1/PlGF ratio was available to clinicians), initial hospitalization rates were much reduced (Figure 2): 76% of women had a sFlt-1/PlGF ratio of ≤38, and only 2% of these women were hospitalized; 11% had a sFlt-1/PlGF ratio of >38-<85, of whom 55% were hospitalized; and 13% had a sFlt-1/PlGF ratio of ≥85, of whom 65% were hospitalized. Overall hospitalization rates were reduced in the test vs. the no-test scenario, with 822 (14%) vs. 1160 (19%) patients hospitalized, respectively (Table 4).

### 3.2. Cost Analyses

Our model demonstrates that additional information provided by the sFlt-1/PlGF ratio may result in clinical management decisions for women with suspected preeclampsia that are better correlated with preeclampsia outcomes than current diagnostic methods alone. The reduction in hospitalization in the test scenario would result in a cost saving of €346 per patient (Table 4). Additional costs of the test (+€856,627) were offset by savings from reduced hospitalization in the test scenario; the total saving in medical costs was €2,962,929 for the test scenario compared with the no-test scenario. Due to the increased number of women receiving outpatient management in the test scenario, costs for low and intermediate care were increased compared with the no-test scenario. In the test scenario, only a small proportion of patients were initially hospitalized and later received outpatient care. Furthermore, hospitalization rates were decreased for patients receiving both low and intermediate outpatient managements, from 15% in the no-test scenario to 4% in the test scenario (Figures 1 and 2), thus demonstrating a decrease in the rate of false-negative preeclampsia diagnosis. Substantial cost savings were therefore obtained with the test scenario compared with the no-test scenario (€13,504,499); this largely balanced the increase in other low/intermediate outpatient management costs. Hospitalization costs for women with suspected preeclampsia who gave birth and women with preeclampsia who initially received ambulatory care were reduced by a total of €2,852,507 in the test scenario.

Based on the assumption that there would be greater implementation of testing over time, budget impact analysis revealed steadily increasing cost savings in the test scenario compared with the no-test scenario, from €421,330 in the first year to €1,835,520 in the fifth year (discounted by 3.5%; Table 5). Total cumulative cost saving over 5 years was €5,867,441, indicating the cost-saving potential of the test scenario (SOC+sFlt-1/PlGF) for the Swiss healthcare system compared with the no-test scenario (current practice).

### 3.3. Sensitivity Analyses

**Figure 3:**
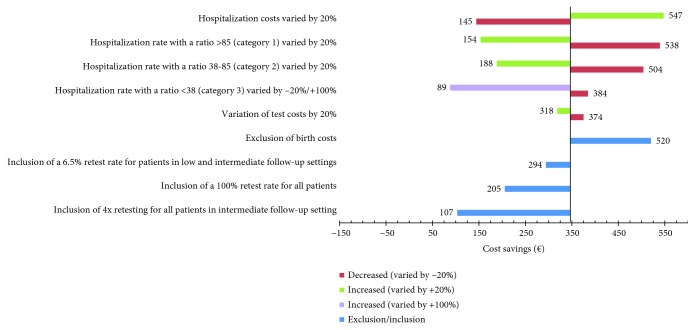
Sensitivity analysis of the impact of hospitalization rate, test cost variation (±20%), exclusion of birth costs, and retest rates on cost savings.

Sensitivity analyses supported the robustness of the value of evaluating the sFlt-1/PlGF ratio in terms of reducing costs when managing women with suspected preeclampsia in Switzerland (Figure 3). The greatest increase in cost savings was demonstrated by increasing hospitalization costs by 20%, resulting in a saving of €547 per patient. Decreasing hospitalization of patients with a sFlt-1/PlGF ratio of ≥85 or a ratio of >38-<85 by 20% produced increased savings of €538 and €504, respectively. The greatest decrease in cost savings was demonstrated by increasing hospitalization of patients with a sFlt-1/PlGF ratio of ≤38 by 100%, resulting in a cost saving of €89 per patient. The exclusion of birth costs produced a saving of €520 per patient. Varying test costs by ±20% had the least impact on cost savings. Cost savings were greater with the inclusion of a 6.5% retest rate (PreOS) for women in the low outpatient setting compared with retesting all women or 4 times retesting of all intermediate follow-up patients (€294 vs. €205 or €107, respectively).

## 4. Discussion

This health economic analysis demonstrates that information provided by evaluating the sFlt-1/PlGF ratio may allow for a more cost-effective management of women with suspected preeclampsia in Switzerland. Our model found an expected cost saving of €346 per patient and a potential annual saving of €2,105,064, supporting the incorporation of sFlt-1/PlGF ratio evaluation into standard practice for the Swiss healthcare system. This estimate may be compared with cost savings previously determined for the UK, Germany, and Italy (€344, €361, and €670 per patient, respectively), each also derived from patient-level data reported in the PROGNOSIS study [14–16]. Differences in cost savings estimated in these analyses may be attributed largely to variations in the healthcare and payer systems used in these European countries.

A high variance in the incidence of preeclampsia has been observed previously among countries and regions, with rates of 1.4-4.0% reported for populations in Australia, Northern Europe, and the USA/Canada [26] and a rate of 4.6% reported worldwide (95% uncertainty range: 2.7-8.2%) [27]. In Switzerland, in particular, the incidence of preeclampsia has been reported to be 2.3% [3]. In our study, we assumed an incidence of 7% for all cases of *suspected* preeclampsia, including patients who do not eventually develop the condition. This rate was based on German guidelines advising that 6-8% of all pregnant women develop hypertension during pregnancy [18].

Our study provides an important insight into the length of hospitalization for patients with suspected preeclampsia in Switzerland, suggesting that these patients are hospitalized longer than necessary. Among the 36 women with suspected but not manifested preeclampsia in the cost analysis, only three were discharged after 24 hours. At present, there are no Swiss guidelines for how patients with suspected preeclampsia should be treated and managed. The main recommendations for the prevention and treatment of preeclampsia provided by the World Health Organization and clinical experts include the use of calcium supplements (excluding developed countries), aspirin, and antihypertensives, as well as interventions, such as induction of labor and early delivery [28, 29].

Since July 2019, reimbursement is available for evaluating the sFlt-1/PlGF ratio in Switzerland. It can be assumed that the costs of hospitalization and medical services would be reduced if the test is established in routine practice (i.e., when doctors rely on the test result), and patient management can be optimized. This is reflected in the results of our analysis, in which cost savings for the test scenario increase over time. Furthermore, sensitivity analysis demonstrated that our base-case cost assumptions are robust to changes in several key parameters. Results were found to be sensitive to variations in hospitalization costs, test costs, and hospitalization rates by the sFlt-1/PlGF ratio. Greater cost savings could be achieved if actual hospitalization costs were higher or if hospitalization rates for patients with sFlt-1/PlGF ratios of ≥85 or >38-<85 could be further reduced. Reduced hospitalization rates for women with an sFlt-1/PlGF ratio of ≤38 could produce the largest cost savings, however; at present, a 100% increase in hospitalization for this group could result in a saving of €89 per patient. This information may be beneficial for clinicians looking to increase cost-effectiveness in treatment management for women with suspected preeclampsia. Whilst the inclusion of a 100% retest rate reduced cost savings by €141 and 4 times retesting of all intermediate follow-up patients reduced cost savings by €239, the inclusion of a 6.5% retest rate (derived from PreOS data for women in the low outpatient setting) only reduced cost savings by €52. This latter approach may provide a more attractive option for Swiss clinicians looking to implement retesting into treatment management for women with suspected preeclampsia, provided that test results are available within a useful timeframe. As the Swiss healthcare system is based on DRG rates for each case, cost savings occur as a result of reduced hospitalization rates rather than a reduction in the use of specific medical interventions or resources for the management of preeclampsia (similar to the German system). In Switzerland, ambulatory care (Tarmed) is paid entirely by health insurance companies, whilst hospitalization is partly covered by the government (55%) [30]. Hence, cost savings due to reduced hospitalization mainly benefit individual cantons, rather than health insurance companies.

The negative prognostic value (NPV) of angiogenic markers to rule out preeclampsia is high (99.3%); however, the positive predictive value (PPV) to rule in preeclampsia is much lower (36.7%). Nevertheless, the PPV of sFlt-1/PlGF is still favourable compared with the current gold standard (blood pressure measuring and proteinuria) which has a PPV of approximately 20% [31]. Additional studies are needed to potentially improve the PPV of the biomarkers for ruling in preeclampsia. Whilst all economic analyses are subject to limitations and are based on assumptions, a major strength of the present study is that real-world cost data were utilized from Swiss hospitals; therefore, our model is likely to be reflective of the clinical practice and management approaches used in Switzerland today. Furthermore, this is the first analysis to assess the cost of preeclampsia patients in Switzerland. Notably, the analysis included birth costs; by excluding them, total costs would have been lower (as seen the in the UK [14] and German studies [15]). However, the inclusion of birth costs (based on registry values), which differ for patients with or without preeclampsia, provides a more complete picture of the impact of preeclampsia on the Swiss healthcare system.

A limitation of this analysis was that the PROGNOSIS protocol specified that women should be admitted to a hospital if their blood pressure exceeded 160/110 mmHg. In clinical practice, a blood pressure of 150/95 mmHg may be considered the threshold for hospital admission, as indicated in the German guidelines [18]. Hence, in practice, rates of hospitalization may be higher, which will also have an associated economic impact. In part, we addressed this point in the sensitivity analyses by variation of hospitalization rates. A further limitation is that costs of neonatal care have not been included; Stevens et al. (2017) demonstrated that the cost burden for neonates exceeded that of their mothers in the 12 months following birth when costs associated with preeclamptic pregnancies were separated into maternal and infant cost episodes by gestational age [32]. Costs of neonatal care may be important to consider in future economic models of this type. Furthermore, costs of anticoagulants and corticosteroids have also not been included. Previous studies have demonstrated that antenatal steroid therapy can reduce the cost of medical services and length of hospitalization for preterm infants [33]. The inclusion of these costs in subsequent models may therefore increase their accuracy.

## 5. Conclusions

The introduction of sFlt-1/PlGF ratio evaluation into Swiss hospital practice appears to be economically promising to predict the short-term absence of preeclampsia. The improvement in diagnostic accuracy and a reduction in unnecessary hospitalization would likely lead to substantial cost savings in the Swiss healthcare system.

## Figures and Tables

**Table 1:** Treatment scenario and services provided for the outpatient setting (low and intermediate follow-up).

Service	Initial consultation (all patients)	Outpatient management	
		Low follow-up	Intermediate follow-up
Duration	—	8 weeks	8 weeks^∗^
Consultation	1x	Every 7-10 days (6x)	Weekly (8x)
Blood pressure measurement	1x	Every 7-10 days (6x)	Weekly (8x)
Blood analyses (ALAT/GPT, ASAT/GOT, LDH, haptoglobin, creatinine)	1x	—	Weekly (8x)
Fetal ultrasound with Doppler/CTG	1x	Every 7-10 days (6x)	Weekly (8x)
Proteinuria (quantitative)	1x	—	—
Proteinuria (fast strip)	—	Every 7-10 days (6x)	Weekly (8x)
sFlt-1/PlGF ratio	1x	—	—
Costs per week (€)	531	200	547

^∗^Based on the assumption that a proportion of women would be hospitalized after 4 weeks. ALAT/GPT: alanine aminotransferase; ASAT/GOT: aspartate aminotransferase; CTG: cardiotocography; LDH: lactate dehydrogenase; PlGF: placental growth factor; sFlt-1: soluble fms-like tyrosine kinase-1.

**Table 2:** Cost of medical services provided in the outpatient setting: initial appointment (all) and follow-up of mild hypertonic pregnant women managed by clinicians.

Service^∗^	Initial appointment (€)	Outpatient management	
		Low follow-up (€)	Intermediate follow-up (€)
Consultation	51.01	51.01	51.01
Gynecologic examination	24.75	24.75	24.75
Preliminary discussion for diagnostic/therapeutic interventions	NP	NP	29.15
Special gynecologic counseling	NP	NP	29.15
Informal report (11-35 lines)	NP	NP	32.05
Document review (patient not present, 18 min)	NP	NP	52.44
Blood pressure measurement	19.60	19.60	NP
Venipuncture for blood withdrawal	6.42	6.42	6.42
Proteinuria (fast strip)	NP	4.58	NP
Proteinuria (quantitative)	141.86	NP	NP
Urine part status (5-10 parameter)	NP	NP	0.88
Thrombocyte, hemoglobin, hematocrit	7.92	NP	NP
ALAT/GPT	2.20	NP	2.20
ASAT/GOT	6.95	NP	2.20
LDH	2.20	NP	2.20
Bilirubin	6.95	NP	NP
Urate	6.95	NP	NP
Creatinine	6.95	NP	2.20
Haptoglobin	17.51	NP	17.51
Blood coagulation test	25.08	NP	NP
Sonography (with fetal Doppler)	58.36	58.36	58.36
Ultrasound examination	79.06	79.06	151.95^‡^
CTG	66.83	22.28	66.83
Total cost per consultation	524.19	259.64	546.94
Total cost per week	NP	199.54^†^	546.94

^∗^Service costs are based on Tarmed v.1.09 and Analysenliste v.2.01 tariffs. ^†^Assuming 6x per patient within 8 weeks of follow-up. ^‡^Extended ultrasound provided during intermediate follow-up. ALAT/GPT: alanine aminotransferase; ASAT/GOT: aspartate aminotransferase; CTG: cardiotocography; LDH: lactate dehydrogenase; NP: not performed.

**Table 3:** Inpatient costs based on two Swiss hospitals (Lucerne Cantonal Hospital and Basel University Hospital [16, 17]).

Indication	N (%)	Effective length of stay, median (days)	Effective length of stay, mean (days)	Median cost based on effective costs (€)^∗^	Mean cost based on effective costs (€)^∗^	Total costs (€)
Suspected preeclampsia without birth	36^†^ (12)	3.0	4.5	5,225	6,300	
Vaginal birth with preeclampsia	101 (34)	4.0	5.4	8,321	10,715	
C-section birth with preeclampsia	164 (54)	6.0	7.8	14,010	17,094	
All	301^‡^ (100)	5.0	6.6	8,102	9,504	4,112,399

^∗^Inpatient costs were derived from registries for each hospital. ^†^Of the 36 patients with suspected preeclampsia who did not give birth, three were admitted for only 24 hours. ^‡^Seven women were excluded due to hospitalization after birth or abortion.

**Table 4:** Comparison of costs for the no-test and test (sFlt-1/PlGF) strategies and difference in total and per patient costs.

Service	No-test strategy		Test strategy		Difference (€)
	*N*	Cost (€)	*N*	Cost (€)	
Initial consultation	6084	3,228,220	6084	3,228,220	—
Outpatient care					
Low	1655	13,068,335	2185	17,248,215	+4,179,880
Intermediate	1655	17,452,138	2498	26,666,335	+9,214,197
Intermediate (after hospitalization)	1613	22,116,244	580	8,611,745	–13,504,499
Hospitalization					
Suspected preeclampsia with birth	583	4,906,013	380	3,191,864	–1,714,150
Preeclampsia after ambulatory care	577	5,698,412	442	4,560,054	–1,138,357
Total medical costs	6084	66,469,362	6084	63,506,433	–2,962,929
sFlt-1/PlGF evaluation	0	—	6084	856,627	+856,627
Overall costs					
Total	6084	66,469,362	6084	64,363,060	–2,106,301
Per patient	6084	10,925	6084	10,579	–346

PlGF: placental growth factor; sFlt-1: soluble fms-like tyrosine kinase-1.

**Table 5:** Budget impact analysis for the no-test and test (sFlt-1/PlGF) strategies.

Year	No-test strategy		Test strategy		Total cost for entire cohort (€)		Difference in costs (no-test strategy vs. test strategy; €)
	*N*	Costs (€) [19]	*N*	Costs (€)	Test/no-test strategy	No-test strategy	
1	4867	53,173,304	1217	12,874,728	66,048,032	66,469,362	–421,330
2	3484	38,063,651	2600	27,505,581	63,351,915	64,221,605	–869,690
3	2434	26,592,115	3650	38,613,604	60,870,237	62,049,860	–1,179,623
4	1084	11,842,996	5000	52,895,349	58,390,277	59,951,556	–1,561,278
5	0	—	6084	64,363,060	56,088,688	57,924,209	–1,835,520
Total within 5 years	11,869	129,672,066	18,551	196,252,323	304,749,152	310,616,592	–5,867,441

PlGF: placental growth factor; sFlt-1: soluble fms-like tyrosine kinase-1.

## Data Availability

The clinical data supporting this health economic analysis are from previously reported studies and datasets, which have been cited. The aggregated economic data used to support the findings are available from the corresponding author upon request.

## Supplementary Materials

Supplementary Materials Search terms used within the accounting departments of Lucerne Cantonal Hospital and Basel University Hospital to determine the actual cost of managing patients with preeclampsia for 2016.
