# Supplementary material for: sFlt-1/PlGF Ratio as a Predictive Marker in Women with Suspected Preeclampsia: An Economic Evaluation from a Swiss Perspective
Source: Dis Markers. 2019 Aug 14;2019:4096847. doi: 10.1155/2019/4096847 (PMC6710794; doi:10.1155/2019/4096847)
Supplement: Supplementary Materials — Search terms used within the accounting departments of Lucerne Cantonal Hospital and Basel University Hospital to determine the actual cost of managing patients with preeclampsia for 2016. [file 4096847.f1.docx]

**Supplementary Material**

Supplementary Table 1: Search terms used within the accounting departments of Lucerne Cantonal Hospital and Basel University Hospital to determine the actual cost of managing patients with preeclampsia for 2016.

| Code | Search term |
| --- | --- |
| O01A | Cesarean section with several complicated diagnoses, duration of pregnancy <25 weeks of gestation or with intrauterine therapy |
| O01B | Cesarean section with several complicated diagnoses, duration of pregnancy: ≥26-≤33 weeks of gestation, <25 weeks of gestation or thromboembolism during the gestation period with operating room procedure or complex procedure |
| O01C | Cesarean section with several complicated diagnoses; duration of pregnancy: >33 weeks of gestation without intrauterine therapy or with complicated diagnosis; ≥26-≤33 weeks of gestation or with complicated diagnosis or with extreme severe complications |
| O01D | Cesarean section with several complicated diagnoses, duration of pregnancy >33 weeks of gestation without intrauterine therapy or with complicated diagnosis, ≥26-≤33 weeks of gestation or with complicated diagnosis or <33 weeks of gestation or with complicated diagnosis without extreme difficult complications |
| O01E | Cesarean section with complicated diagnosis, duration of pregnancy >33 weeks of gestation without complex diagnosis |
| O02B | Vaginal delivery with complicated operating room procedure |
| O05B | Specific operating room procedures during pregnancy or intrauterine therapy of fetus |
| O60A | Vaginal delivery with several complicated diagnoses, at least one severe, duration of pregnancy <33 weeks of gestation or with complicated procedure or thromboembolism during the gestation period |
| O60B | Vaginal delivery with several complicated diagnoses, at least one severe, duration of pregnancy >33 weeks of gestation, without complicated procedure or thromboembolism during the gestation period |
| O60C | Vaginal delivery with severe or mild complicated diagnosis |
| O60D | Vaginal delivery |
| O61Z | Hospitalization after delivery or abortion without operating room procedure |
| O65A | Other prenatal hospitalization with severe complications or complex diagnosis, duration of pregnancy 20-33 weeks of gestation |
| O65B | Other prenatal hospitalization with severe complications or complex diagnosis, duration of pregnancy <19 or >33 weeks of gestation |
| O65C | Other prenatal hospitalization, duration of pregnancy <26 or >33 weeks of gestation |
| *O65D* | Other prenatal hospitalization, one day of occupancy |
| I12* | Hypertensive renal disease |
| I15* | Secondary hypertonia |

*No patients found using these searches.
